# Relationship Between Length and Curvature of Ascending Aorta and Type a Dissection

**DOI:** 10.3389/fcvm.2022.927105

**Published:** 2022-06-20

**Authors:** Lianjie Sun, Xiao Li, Guoqing Wang, Jianchao Sun, Xiaoming Zhang, Honghui Chi, Huihui Cao, Wanteng Ma, Zhisheng Yan, Gaoli Liu

**Affiliations:** ^1^Department of Cardiovascular Surgery, The Affiliated Hospital of Qingdao University, Qingdao, China; ^2^Department of Ultrasound in Medicine, Shanghai Jiao Tong University Affiliated 6th People's Hospital, Shanghai Institute of Ultrasound in Medicine, Shanghai, China

**Keywords:** aortic dissection, aneurysm, aortic elongation, aortic morphology, computed tomography

## Abstract

**Background:**

Type A aortic dissection (TAAD) has a rapid onset and high mortality. Currently, aortic diameter is the major criterion for evaluating the risk of TAAD. We attempted to find other aortic morphological indicators to further analyze their relationships with the risk of type A dissection.

**Methods:**

We included the imaging and clinical data of 112 patients. The patients were divided into three groups, of which Group 1 had 49 patients with normal aortic diameter, Group 2 had 22 patients with ascending aortic aneurysm, and Group 3 had 41 patients with TAAD. We used AW Server software, version 3.2, to measure aorta-related morphological indicators.

**Results:**

First, in Group 1, the univariate analysis results showed that ascending aortic diameter was correlated with patient age (*r*^2^ = 0.35) and ascending aortic length (AAL) (*r*^2^ = 0.43). AAL was correlated with age (*r*^2^ = 0.12) and height (*r*^2^ = 0.11). Further analysis of the aortic morphological indicators among the three groups found that the median aortic diameter was 36.20 mm in Group 1 (Q1–Q3: 33.40–37.70 mm), 42.5 mm in Group 2 (Q1–Q3: 41.52–44.17 mm) and 48.6 mm in Group 3 (Q1–Q3: 42.4–55.3 mm). There was no significant difference between Groups 2 and 3 (*P* > 0.05). Group 3 had the longest AAL (median: 109.4 mm, Q1–Q3: 118.3–105.3 mm), followed by Group 2 (median: 91.0 mm, Q1–Q3: 95.97–84.12 mm) and Group 1 (81.20 mm, Q1–Q3: 76.90–86.20 mm), and there were statistically significant differences among the three groups (*P* < 0.05). The Aortic Bending Index (ABI) was 14.95 mm/cm in Group 3 (Q1–Q3: 14.42–15.78 mm/cm), 13.80 mm/cm in Group 2 (Q1–Q3: 13.42–14.42 mm/cm), and 13.29 mm/cm in Group 1 (Q1–Q3: 12.71–13.78 mm/cm), and the difference was statistically significant in comparisons between any two groups (*P* < 0.05). Regression analysis showed that aortic diameter + AAL + ABI differentiated Group 2 and Group 3 with statistical significance (area under the curve (AUC) = 0.834), which was better than aortic diameter alone (AUC = 0.657; *P* < 0.05).

**Conclusions:**

We introduced the new concept of ABI, which has certain clinical significance in distinguishing patients with aortic dissection and aneurysm. Perhaps the ascending aortic diameter combined with AAL and ABI could be helpful in predicting the occurrence of TAAD.

## Introduction

TAAD is an acute aortic adverse event (AAE) with rapid onset and a high fatality rate. One-fifth of patients die without emergency treatment (1, 2), and the mortality rate increases by 1–2% every hour (3, 4). Surgery should be performed immediately after diagnosis, but emergency surgery mortality is still high (5), prevention is particularly important.

Ascending aortic aneurysm, a focal dilation of the ascending aorta, is the main cause of TAAD. Replacing the diseased blood vessel before it ruptures is the main preventive measure, greatly reducing the incidence and mortality of TAAD. Currently, the diameter of the ascending aorta is the only indication for surgical evaluation, and the guideline recommends 5.5 cm as an indication for surgery in ascending aortic aneurysm (6). However, studies have found that up to 50% of Type A dissections occur in vessels with diameters less than the surgical threshold (7, 8). The latest study by a Yale team suggested lowering the diameter threshold to 5 cm (9). Relying solely on aortic diameter to formulate surgical indications might not be perfect, and it is particularly important to look for other indicators.

We measured ascending aortic diameter, AAL and other aortic morphological indicators by computed tomographic angiography (CTA) imaging and additional analysis, attempting to identify other risk factors for TAAD.

## Methods

### Participants

We reviewed clinical data from the Affiliated Hospital of Qingdao University between January 2020 and December 2021, and a total of 362 patients had thoracic aortic CTA data. The exclusion criteria were as follows: (1) poor imaging quality and lack of preoperative CTA; (2) hematoma or type B aortic dissection; (3) iatrogenic, traumatic dissection, and previous iatrogenic procedures that could damage the ascending aorta, such as ascending aortic cannulation and bypass; (4) Marfan's syndrome and Behcet's disease; (5) age <18 years old or lack of basic data, such as height; (6) aortic malformation.

A total of 112 patients were eventually included. There were 67 men and 45 women with an age range of 26–89 years old and a mean age of 60.97 ± 15.08 years old. The protocol was approved by the Qingdao University Ethics Committee, and it conformed to the Declaration of Helsinki.

### Aortic Morphological Examination

We used AW Server software, version 3.2 (General Electric Company), to further process the CTA data. The images were retrospectively reviewed by three experienced physicians. When interpretations differed, the majority opinion was used.

#### Aortic Diameter

We measured the sino-tubular junction (STJ) plane, the proximal innominate artery plane, and the maximum diameter of the ascending aorta.

#### AAL and ABI

**Figure 1:**
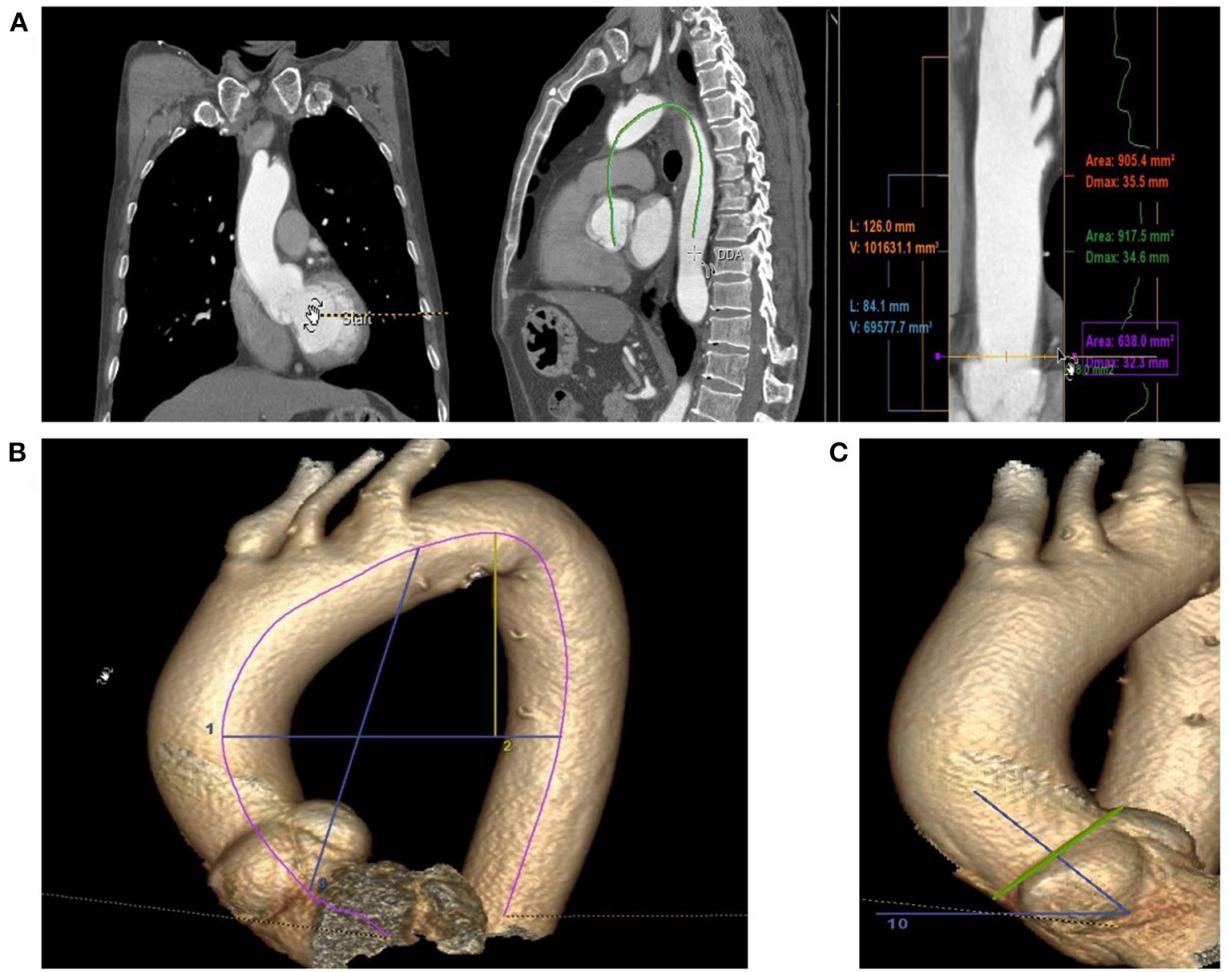
The methods of Aortic morphological examination; **(A)** Markers are placed from the aortic root to the furthest distal end of the visible aorta, ensuring accurate coverage of the aortic arch and the measurement of Ascending Aorta Diameter and Length. **(B)** Measurement of ABI and HWR; the line 8: Straight line distance from the aortic root to the opening of left subclavian artery; line 2: the maximal horizontal distance between the midpoints of the ascending and descending aorta close to the axial plane; line 1: the maximal vertical distance between line 2 and the highest midpoint of the aortic arch. **(C)** The angle between the vertical line of the STJ plane and the horizontal plane.

Markers were placed from the aortic root to the furthest distal end of the visible aorta, ensuring accurate coverage of the aortic arch. Semiautomatic centerlines were generated through the entire length of the aorta, and we manually checked for proper symmetry around the centerline. For the AAL measurement, markers were manually placed at the annulus and proximal limit of the origin of the innominate artery to define the ascending aorta (Figure 1A). Since dissection increases the diameter and length (10–12), the aortic diameter and length were corrected according to Wu's model, and we also measured the length (l) and the straight line distance (d) from the aortic root to the left subclavian artery. The ratio of the above two was ABI, defined as ABI = l/d, used to assess the degree of curvature of the ascending aorta (Figure 1B).

#### Height-to-Width Ratio (HWR)

The width of the aortic arch (W) was measured as the maximal horizontal distance between the midpoints of the ascending and descending aorta close to the axial plane going through the right pulmonary artery. The height of the aortic arch (H) was measured as the maximal vertical distance between W and the highest midpoint of the aortic arch (Figure 1B) (13, 14).

#### Aortic Angle

The aortic angle is the angle between the vertical line of the STJ plane and the horizontal plane. In a few patients, the body was inclined during the examination, and we corrected for this position according to the angle between the connecting line of the corresponding rib on the coronal plane and the horizontal plane (Figure 1C).

#### The Morphology of the Aortic Arch

We also documented the morphology of the aortic arch. In the type I arch configuration, the apex and reversal point of the arch are proximal to the left subclavian artery (LSA); and in the type II arch configuration, this point is distal to the left subclavian artery (15, 16).

### Statistical Analysis

Statistical analyses were performed with the R software, version 4.1.2. The normality of distribution was checked with Kolmogorov–Smirnov test. Normally distributed data are expressed as the mean ± standard deviation, whereas data not normally distributed are expressed as the median and interquartile range. Categorical variables were expressed as percentages. Independent two-sample *t*-test and non-parametric test (Wilcoxon) were used for comparison of quantitative data. Pearson's chi-square (χ^2^) tests were used to compare categorical data. Univariate and multivariate analyses were performed to identify aortic morphology-related factors associated with type A aortic dissection or aneurysm. Random forest regression was used to build predictive models. Receiver operating characteristic (ROC) curves were used to obtain their AUCs, and Delong's test was used to test the AUC significance. A *p* < 0.05 indicated statistical significance.

## Results

### Baseline Characteristics

There were 112 patients included in the study, including 67 men and 45 women, with an average age of 65 ± 15.08 years old. According to the occurrence of TAAD, the patients were divided into a non-dissection group and a TAAD group (Group 3). The non-dissection group was further divided into an aneurysm group (Group 2) and a normal diameter group (Group 1) according to aortic diameter. Information about the clinical data among the three groups is shown in Table 1.

**Table 1:** Clinical data.

**Parameter**	**Group 1 (*n* = 49)**	**Group 2 (*n* = 22)**	**Group 3 (*n* = 41)**	***P*** **-value**
N (M/F)	49 (28/21)	22 (15/7)	41 (24/17)	>0.05
Age (years)	63.4 ± 16.8_a_	66.3 ± 13.0_a_	55.2 ± 12.2_b_	<0.05
Height (cm)	165.9 ± 7.3	167.6 ± 7.9	168.6 ± 7.4	>0.05
Weight (kg)	67.6 ± 10.6_a_	73.9 ± 13.7_ab_	77.5 ± 13.6_b_	<0.05
BMI (kg/m^2^)	24.5 ± 2.8_a_	25.2 ± 6.7_b_	27.2 ± 3.7_b_	<0.05
Aortic valve disease (%)	8 (16.7%)	4 (18.2%)	7 (17.0%)	>0.05
Hypertension (%)	26 (55.3)_a_	17 (77.3%)_ab_	31 (75.6%)_b_	>0.05
CHD (%)	18 (40.0%)_a_	11 (52.4%)_a_	4 (9.4%)_b_	<0.05
Renal insufficiency (%)	2 (4.3%)	0 (0.0%)	2 (4.8%)	-
BAA (%)	2 (4.3%)	4 (18.1%)	1 (2.4%)	-
Smoke (%)	14 (29.2%)	5 (22.7%)	9 (22.5%)	>0.05

*Data are shown as the mean ± SD or the median (interquartile range). M/F, male/female; BMI, body mass index; CHD, coronary atherosclerotic disease; BAA, bovine aortic arch. Subscript letters indicate that at the 0.05 level, the column proportions for these categories were not significantly different from each other*.

### Normal Ascending Aortic Morphology

**Figure 2:**
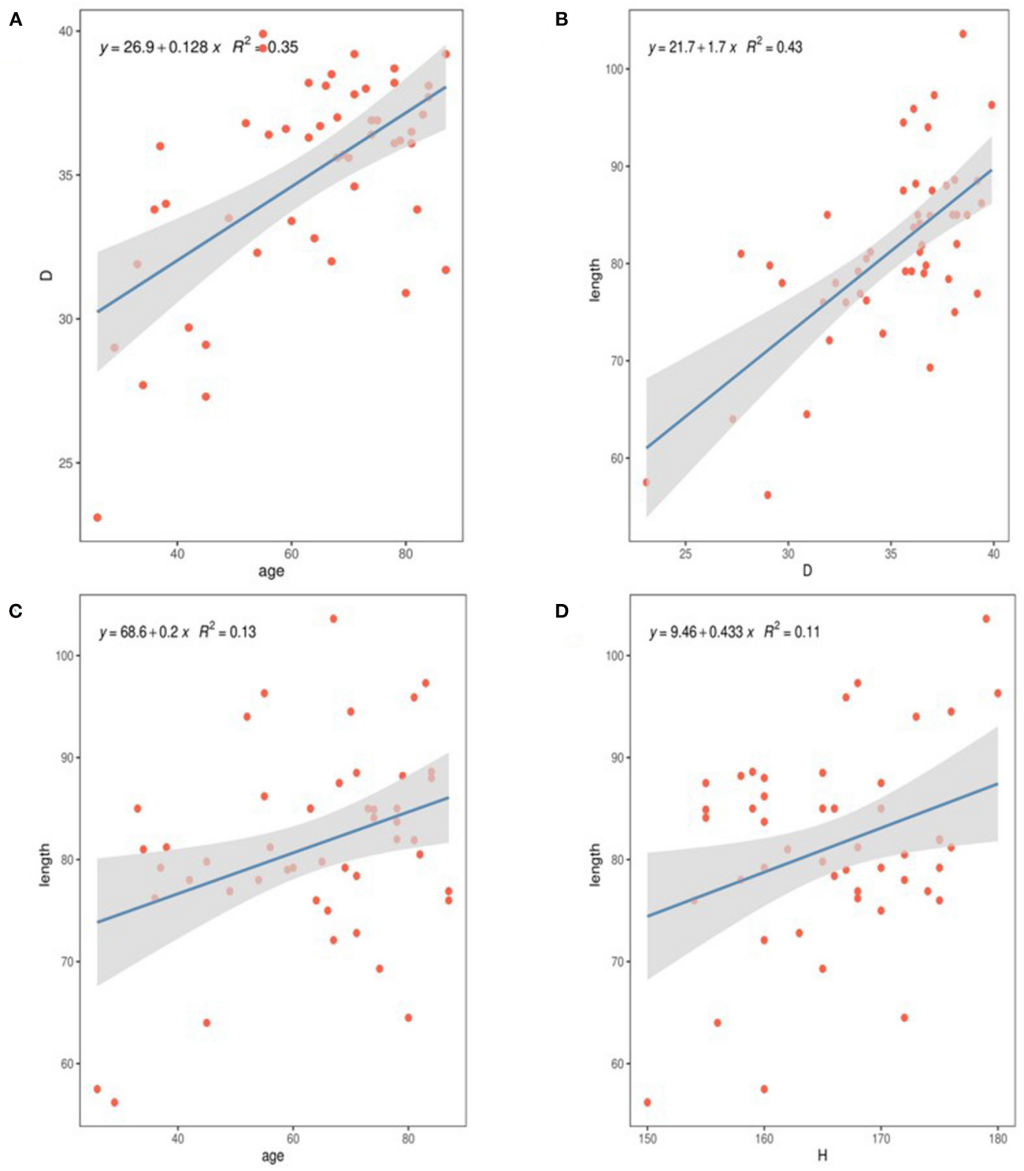
The relationship of diameter and length in normal ascending aortas with clinical variables. **(A)** The diameter of the ascending aorta correlates with increasing age. **(B)** The diameter of the ascending aorta correlates with increasing AAL. **(C)** The AAL was correlates with increasing age. **(D)** The AAL was correlates with increasing height.

We measured the diameter of STJ at a median of 30.60 mm (Q1–Q3: 28.60–29.96 mm), the innominate artery at 34.30 mm (Q1–Q3: 31.40–36.30), the largest diameter of the ascending aorta at a median of 36.20 mm (Q1–Q3: 33.40–37.70 mm), and the measured AAL at a median of 81.20 mm, (Q1–Q3: 76.90–86.20 mm). The ABI was a median of 13.29 mm/cm (Q1–Q3: 12.71–13.78 mm/cm), the median height-to-width ratio was 0.531 (Q1–Q3: 0.47–0.60), the median aortic angle was 50.5° (Q1–Q3: 47.1°-55.2°), and a type I arch was found in 49%. There were no significant differences in aortic diameter, length or age between men and women (*P* > 0.05). Aortic diameter was correlated with age (*r*^2^ = 0.35, *p* < 0.01; Figure 2A), and AAL was closely related to diameter (*r*^2^ = 0.43, *p* < 0.05), age (*r*^2^ = 0.12, *P* < 0.05) and height (*r*^2^ = 0.11, *p* < 0.05; Figures 2B–D).

### Ascending Aortic Morphology in Patients With Aneurysms and Dissection

**Figure 3:**
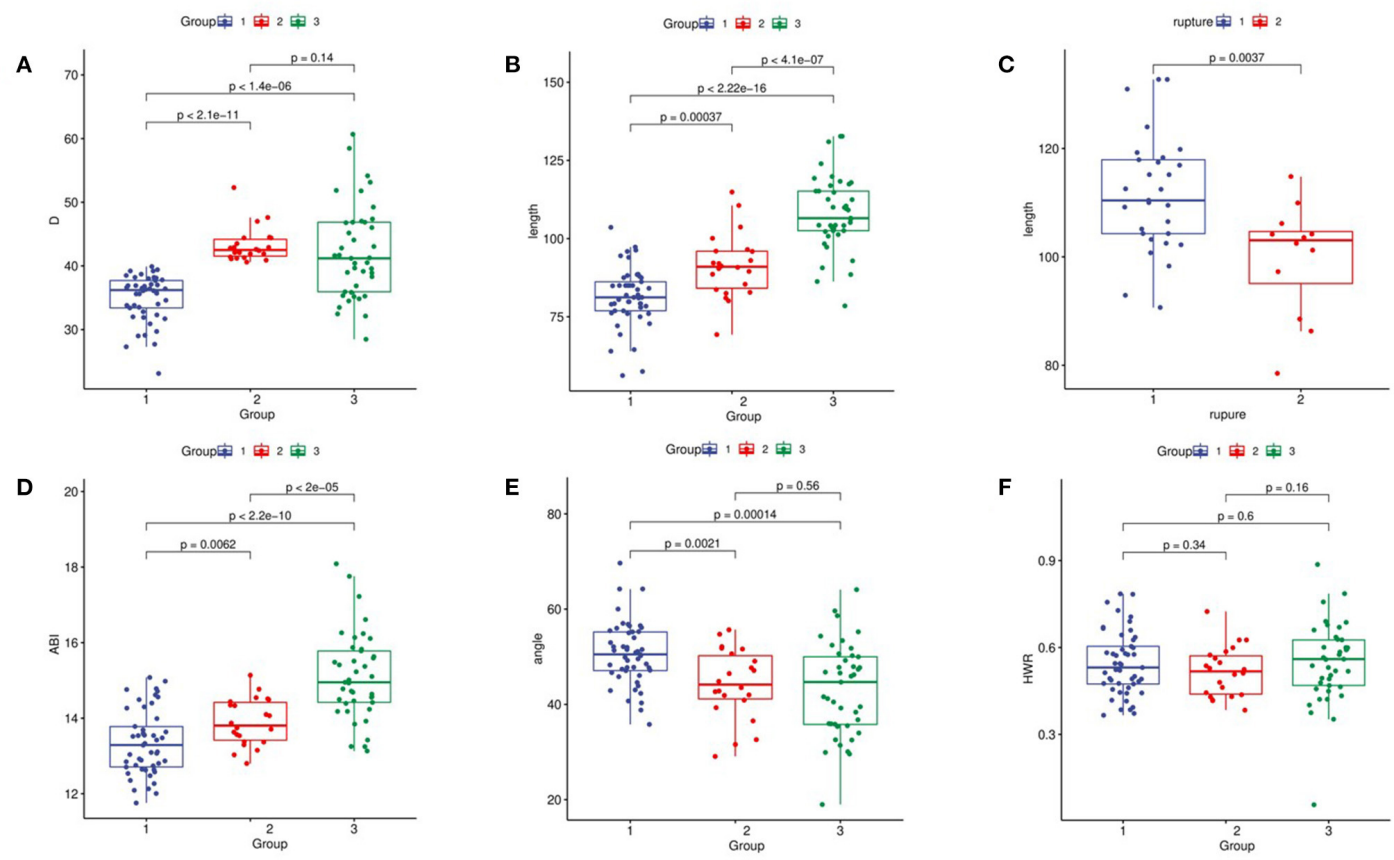
Comparison of ascending aortic morphology. **(A)** Ascending aorta diameter. **(B)** AAL. **(C)** Relationship between aortic length and aortic rupture; rupture 1: the rupture in ascending aorta; rupture 2: the rupture in Distal ascending aorta. **(D)** ABI. **(E)** Ascending aortic angle. **(F)** HWR.

The diameter of the dissection group was 48.6 mm (Q1–Q3: 42.4–55.3 mm), and the aneurysm group had a median diameter of 42.5 mm (Q1–Q3: 41.52–44.17 mm). There was no significant difference (*P* > 0.05). Only 29% of patients in the dissection group had a diameter > 5.5 cm, and approximately 60% of patients had a diameter <5.0 cm. After adjustment (Figure 3A), only 15% of the patients in the dissection group were >5.0 cm, only two cases were >5.5 cm, and approximately 44% of the patients had diameters <4.0 cm.

The AAL in the dissection group was 109.4 mm (Q1–Q3: 118.3–105.3 mm) vs. 91.0 mm in the aneurysm group (Q1–Q3: 95.97–84.12 mm), and the difference among the three groups was statistically significant. After adjustment, the AAL in the dissection group was 106.52 mm. The difference among the three groups remained statistically significant (*p* < 0.05; Figure 3B). AAL > 11 cm was found in 40% of patients in the adjusted dissection group and in only 2% of patients in the aneurysm group. In the dissection group, 70.7% of the patients had an aortic rupture in the ascending aorta, 24.4% were in the aortic arch, and the rest were formed by reverse tearing of the descending aorta. The patients with ruptures in the ascending aorta had a longer ascending aorta (*p* < 0.01; Figure 3C).

The ABI was 14.95 mm/cm (Q1–Q3: 14.42–15.78 mm/cm) in the dissection group vs. 13.80 mm/cm (Q1–Q3: 13.42–14.42 mm/cm) in the aneurysm group, and the difference among the three groups was statistically significant (*P* < 0.01; Figure 3D). Regression analysis showed that ABI was significantly associated with TAAD with a cutoff value of 14.14 mm/cm (95% confidence interval 0.732–0.854).

### Other Indicators

The control group had a greater aortic angle than the dissection group and the aneurysm group (*p* < 0.01), while the difference between the aneurysm group and the dissection group was not statistically significant (*p* = 0.84; Figure 3E). We found that, in the dissection group, nearly 60% had type I arches, while 18% had type I arches in the aneurysm group (*p* < 0.05), compared with 49% in the control group. Hypertension was present in 77.3% of patients in the aneurysm group and 75.6% in the dissection group. There was no statistically significant difference in the height-to-width ratio among the groups (Figure 3F).

### Regression Analysis With Aneurysms and Dissections

**Figure 4:**
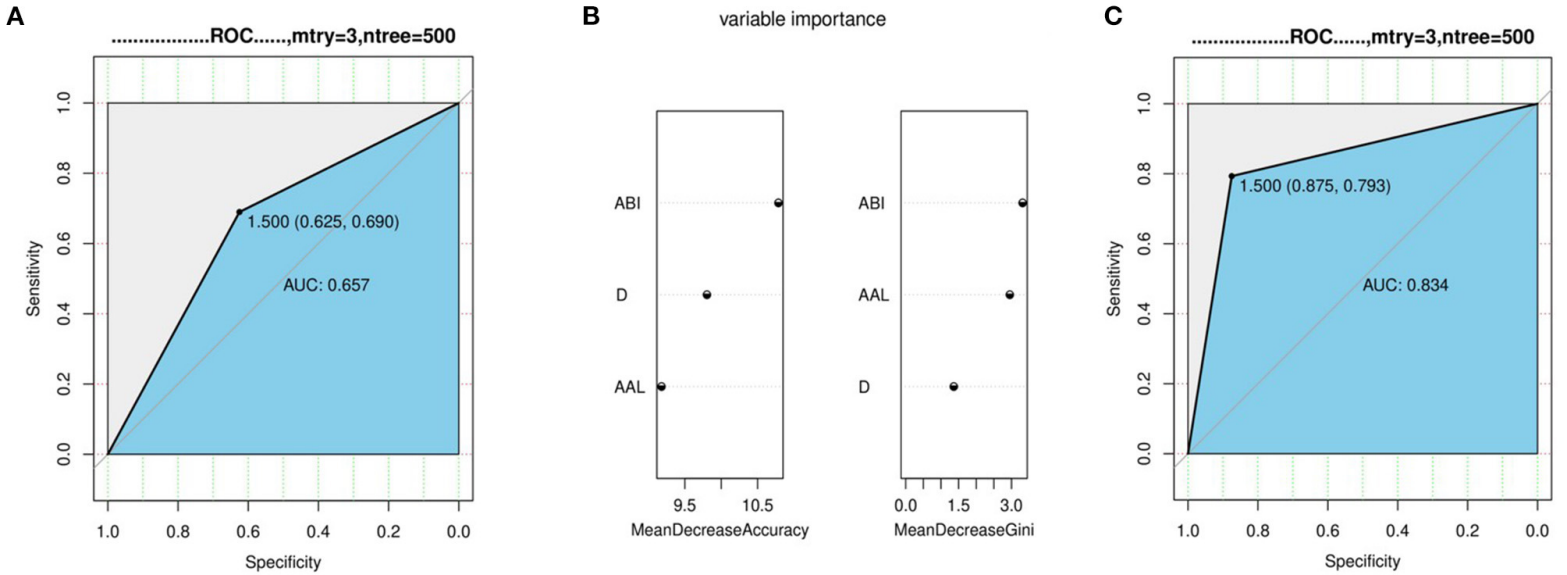
Regression analysis with aneurysms and dissections. **(A)** The ability of diameter distinguish between dissection and aneurysm. **(B,C)** The variable importance and ability of diameter + AAL + ABI distinguish between dissection and aneurysm.

We divided the data of the aneurysm group and the dissection group into a training set and a test set, respectively, at a ratio of 7:3 and incorporated them into the random forest regression model to test the ability of diameter, length, and ABI to distinguish between dissection and aneurysm. A ROC curve was drawn. The simple diameter AUC was 0.657 (Figure 4A), and diameter + AAL + ABI was 0.834 (Figures 4B,C; *p* < 0.05).

## Discussion

In this study, we used the AW Server software system to measure multiple aortic morphological indicators that could lead to aortic dissection (14) and attempted to find new morphological indicators other than diameter alone to better classify patients presenting with aortic dissection from patients with non-dissected aneurysm. We found that there was no significant difference in aortic diameter between the aneurysm group and the dissection group. Even if the surgical indications were relaxed to 5.0 cm, the diameter of more than half of the dissection patients still did not meet the surgical intervention criterion. This phenomenon is known as the aortic size paradox. Paruchuri explained this phenomenon by calculating the relative risk of aortic dissection. People with small aortic diameters have a relatively low risk, but the base of such populations is large, so the absolute number of patients with aortic dissection is high. At the same time, study found that even if the aorta is mildly dilated, the relative risk of dissection increases hundreds of times (7, 17). So it is necessary to look for indicators other than diameter.

Multiple aortic morphological indicators have been studied in the past to eliminate the influence of body size (18) on aortic dimension. Davis AE used diameter/body surface area to express relative aortic size (19), called the aortic size index (ASI), as a predictor of adverse aortic events (AAEs). Based on 2.75 and 4.24 cm/m^2^, the risk of AAE was divided into three grades—low, medium and high—and the predictive ability of ASI was more reliable than that of absolute aortic size. Considering account the instability of adult weight, Zafar et al. (9) proposed the Diameter Height Index (DHI), which uses height alone to correct aortic diameter. The calculation method is diameter (cm)/height (m). Compared with ASI, the accuracy of this index is greater.

With the emergence of new aortic morphological indicators, such as aortic length, Wu et al. (11) used the sum of aortic diameter and length divided by height as a new aortic morphological index, called the aortic height index (AHI), but after dissection, the aortic diameter and length increased, rendering the measurement inaccurate. At the same time, the rupture of type A dissection is not limited to the ascending aorta. We found that 29.3% of the patients had ruptures located far from the opening of the innominate artery, and these patients had smaller ascending aortas (*p* < 0.05), for which we introduced the concept of ABI.

Calculating the ratio of the length and distance from the aortic root to the distal opening of the left subclavian artery reflected the curvature of the ascending aorta-aortic arch, and we found that it was statistically significant among the three groups. The interquartile range of the non-dissection group did not exceed 1.0 mm/cm, which was relatively stable. Dilation and distortion of the aorta will produce an abnormal blood flow state, which will in turn increase the peak wall stress (PWS). Dissection or rupture will occur when PWS exceeds aortic wall strength (20, 21), providing hemodynamic theoretical support for ABI. Random forest regression results showed that the combination of diameter with AAL and ABI has better diagnostic performance than diameter alone to predict the occurrence of dissection.

In our study, the length of the ascending aorta in the adjusted dissection group was 14.5 mm longer than that in the aneurysm group and 25.3 mm longer than that in the control group. The average length of the ascending aorta measured in the control group was 8.1 cm, which was smaller than in previous studies (22), perhaps related to region and ethnicity. There was no significant difference in the aortic dimension between men and women in the control group, which might be related to the patients in the control group being mostly hospitalized patients, the sample size being small, and other cardiovascular diseases not being able to be strictly excluded, as indicated by Pham et al. (23), which could result in insignificant gender differences. The aorta lengthens with age, consistent with previous studies (24, 25), which might be related to the aging of the aorta and the reduction of vascular wall strength due to elastin rupture (26), and according to Laplace's law, the aorta's enlargement itself will further increase the vascular wall tension and accelerate the lengthening of the aorta. In the dissection group, there was no correlation between the length of the aorta and age, and the median age was younger than that in the aneurysm group, perhaps due to the premature aging of the aortic wall tissue in patients with dissection, reducing the strength of the blood vessels and resulting in abnormal dilation of the aorta, which eventually dissolves.

More than 3/4 of patients with dissection have hypertension, and 70% of them are grade 3. Due to the low awareness rate and the low drug use rate for hypertension, some patients must be monitored according to preoperative and postoperative arterial blood pressure during hospitalization to diagnose, but some patients have pericardial effusion or are even in a state of shock when present at the hospital. The prevalence of hypertension might be underestimated, and whether high blood pressure causes aortic dilatation is unclear (14, 27). However, studies have found that high wall stress caused by blood pressure plays an important role in dissection, and aggressive blood pressure control significantly reduces the risk of dissection (21). Unlike previous studies (16), we did not find a correlation between blood pressure and the morphology of the aortic arch.

We measured the angle between the STJ plane and the horizontal plane to estimate the direction of blood flow in the outflow tract. The change of direction could cause abnormal hemodynamic changes and local wall shear stress changes in blood vessels. There was little difference in the dissection and control groups, and there was no significant difference between the dissection and aneurysm groups. Alhafez et al. (13) observed a difference in the aspect ratio in patients with bicuspid aortic valves. We wanted to investigate whether it might play a role in aneurysm progression, but no significant difference was found.

There were several limitations to our study. First, it was a retrospective examination at a single institution. Most of the patients with thoracic aortic aneurysm were asymptomatic, and the data in the control group came mostly from hospitalized patients with cardiovascular diseases, such as hypertension or coronary heart disease, resulting in fewer data in the control and aneurysm groups. Second, because aortic dissection is sudden, obtaining predissection CTA data is difficult, and we adjusted data, such as length and diameter, according to previous research models, which might have caused errors. For this reason, we also used the ABI, a stable index, to represent the degree of aortic curvature. Finally, the aortic growth rate is also an important indicator of the occurrence of AAE requiring long-term follow-up of aneurysm patients and multiple CTA tests, which become difficult in actual clinical practice, and we did not collect data on this aspect.

## Conclusion

We proposed a new concept of ABI, which has certain clinical significance in identifying patients with dissection and aneurysm, and ABI >14.14 mm/cm was a risk factor for occurrence of TAAD. Combining ABI with aortic diameter and AAL might be more useful in predicting the occurrence of TAAD.

## Data Availability Statement

The raw data supporting the conclusions of this article will be made available by the authors, without undue reservation.

## Ethics Statement

Ethical review and approval was not required for the study on human participants in accordance with the local legislation and institutional requirements. Written informed consent for participation was not required for this study in accordance with the national legislation and the institutional requirements.

## Author Contributions

LS drafted the manuscript. XL and HoC performed the statistical analysis. GW, XZ, and JS drafted the figure and legend. HuC and WM wrote sections of the manuscript. GL and ZY designed the outline of the topic and helped on revising the manuscript. All authors contributed to the article and approved the submitted version.

## Funding

The project was funded by National Health Commission (YYWS1918).

## Conflict of Interest

The authors declare that the research was conducted in the absence of any commercial or financial relationships that could be construed as a potential conflict of interest.

## Publisher's Note

All claims expressed in this article are solely those of the authors and do not necessarily represent those of their affiliated organizations, or those of the publisher, the editors and the reviewers. Any product that may be evaluated in this article, or claim that may be made by its manufacturer, is not guaranteed or endorsed by the publisher.
